# Community well-being dimensions in Gunung Mulu National Park, Sarawak, Malaysian Borneo

**DOI:** 10.1057/s41599-023-01737-4

**Published:** 2023-05-09

**Authors:** Mohamad Syahrul Nizam Ibrahim, Shazali Johari, Mohamad Ibrani Shahrimin Adam Assim, Syamsul Herman Mohammad Afandi, Waseem Razzaq Khan, Suziana Hassan

**Affiliations:** 1 Faculty of Agricultural Science and Forestry, Universiti Putra Malaysia Bintulu Campus, Sarawak, Malaysia; 2 Faculty of Forestry and Environment, Universiti Putra Malaysia, Selangor, Malaysia; 3 Faculty of Humanities, Management and Science, Universiti Putra Malaysia Bintulu Campus, Sarawak, Malaysia; 4 School of Business and Economics, Universiti Putra Malaysia, Selangor, Malaysia; 5 Faculty of Economics and Management, Universiti Kebangsaan Malaysia, Selangor, Malaysia

**Keywords:** Environmental studies, Sociology, Geography, Psychology

## Abstract

The local communities living around national parks or areas like World Heritage Site (WHS) are crucial stakeholders to such settings. Their well-being needs to be unraveled so that the holistic management of the national park is in good condition to stabilize its status as WHS through the support and empowerment of the community. Numerous studies have been conducted on the biodiversity and geology of Gunung Mulu National Park (GMNP), but the community psychology aspect that is the foundation of conservation efforts has not been addressed. Therefore, this study aims to examine the community well-being dimensions in terms of environment, economics, social aspects as well as authority intervention based on the perspective of the local community and professionals with an emphasis on the current issues in GMNP. Quantitative and qualitative approaches were used in this study through a questionnaire to 99 local communities, and individual interviews that were conducted in GMNP and four nearby villages. Data were analyzed descriptively with four themes: environment; economics; social; and authority intervention. The findings showed that locals were satisfied in residing area in terms of environmental conditions. However, it does not reflect the actual situation, i.e., river water cloudiness, wildlife threat, degradation of wetlands, and solid waste issues are still happening. The constraints of the COVID-19 pandemic portrayed that they were very dissatisfied with their monthly income, which is very low compared to before. In terms of social, the services and facilities, especially treated water and electricity need improvement. It also noted that authority intervention especially related to road proposal, financial and skills assistance, and community conflicts could influence locals’ support for the planning and policies implemented in the national parks or WHS areas. This study suggests that relevant stakeholders should emphasize bottom-up approaches by considering aspects of community well-being that stem from multiple dimensions in order to achieve holistic national park management.

## Introduction

Well-being is viewed as the state of people’s life conditions (Sumner, 2006) and some researchers have examined community well-being by using individual attributes such as satisfaction, happiness, quality of life, individual efficacy/agency, and/or social support (Andereck et al., 2007; Jurowski and Brown, 2001; Kerstetter and Bricker, 2012). Well-being measurements have progressed to encompass broader dimensions such as social and environmental aspects, and human rights (Sumner, 2006). It is now widely accepted that well-being is a multidimensional concept that encompasses all aspects of human life. Sustainability theories increasingly incorporate utilitarian concepts of well-being, demanding the development of destinations that provide more advantages to a higher number of people within the constraints of available resources (Kay Smith and Diekmann, 2017). The overall welfare of a community necessitates that together these various components work well and maintain a healthy balance (Christakopoulou et al., 2001).

Community well-being is a combination of social, economic, environmental, cultural, and political conditions identified by individuals and their communities as crucial for them to flourish and fulfill their potential (Wiseman and Brasher, 2008). Cummins (1996b) found that the satisfaction associated with the community well-being domain occurs when people satisfied with education, neighborhood, service, facilities, social life, and social relations. Community satisfaction makes a significant and positive contribution to community members’ perceptions of their quality of life (Norman et al., 1997). Many factors can directly or indirectly affect community well-being. Equally, one aspect of community well-being can impact another (Lee and Kim, 2015). For instance, there is a well-established link between economic well-being and health (Bushell and Sheldon, 2009), living environment and psychological needs (Aziz et al., 2022), as well as community satisfaction and attachment to an area (Özkan et al., 2019; Theodori, 2001). Finally, community members with a high level of place attachment are more likely to engage in local volunteer work and collaborate with them and influence the the positive change in the community (Mulaphong, 2022).

In the wider context, human well-being is when individuals able to cope with psychological, social and physical challenges (Dodge et al., 2012). The definition of human well-being is complex and subjective from different perspectives (Clark, 2014). In the context of the local community, well-being will be achieved if they have good satisfaction with the dimensions of the environment, economy, life and social relation, services and facilities, education, neighborhood, and culture, which is in line with some previous studies (Andrews and Withey, 1976; Cummins, 1996a, 1996b; Norman et al., 1997; O’Brien and Lange, 1986; Wiseman and Brasher, 2008). Authority intervention is considered a mediating variable in this study. Current administration and political power determine the well-being of the local population via socioeconomic indicators and infrastructure, while also taking into account their satisfaction with economic, social, and environmental dimensions. For example, if the local community is satisfied with these dimensions, then they would realize the importance of sufficient funds in the effort towards conservation so that they could also enjoy the benefits of a healthy ecosystem such as fresh air, adequate income, good mental and physical health and a healthy source of food. The locals’ scientific and indigenous knowledge of conservation also contributes to a strong sense of physical and spiritual connection to a place with rich natural and cultural attributes (Mokuku and Taylor, 2015). While, locals with low ecocentrism and limited conservation knowledge are more likely to engage in economic activities that disregard sustainability (Kaufman, 2015).

The local community in GMNP is frequently dissatisfied with the local government in regards to land ownership and logging activities that threaten their traditional way of life. This relates back to the notion that human rights are a necessity that safeguards dignity and equality, which is emphasized at the global level (Clark, 2014). Although there are initiatives by the government in improving their standard of living, however, to what extent do they want to accept such initiatives in improving their well-being? Thus, human development, which describes a process of enlarging people’s freedoms and opportunities and improving their well-being can also be challenged. In this light, the importance of empathy rises to the forefront as the primary focus for empowering communities to construct resilience in the face of crises and healing things that drive a wedge between them (Berardi et al., 2020). Communication between the local community and the state government, which leads to understanding and support towards biodiversity conservation efforts at GMNP, needs to be further refined in terms of its effectiveness. Thus, the ground-level issues of the locals need to be addressed so that their well-being can be sustained and become the key to the effective biodiversity framework for Gunung Mulu National Park (GMNP). Therefore, in this study, we aimed to explore the community well-being dimensions in terms of environment, economics, and social aspects as well as authority intervention based on the perspective of the local community and professionals with an emphasis on the current issues in GMNP.

## Conceptual framework

**Fig. 1:**
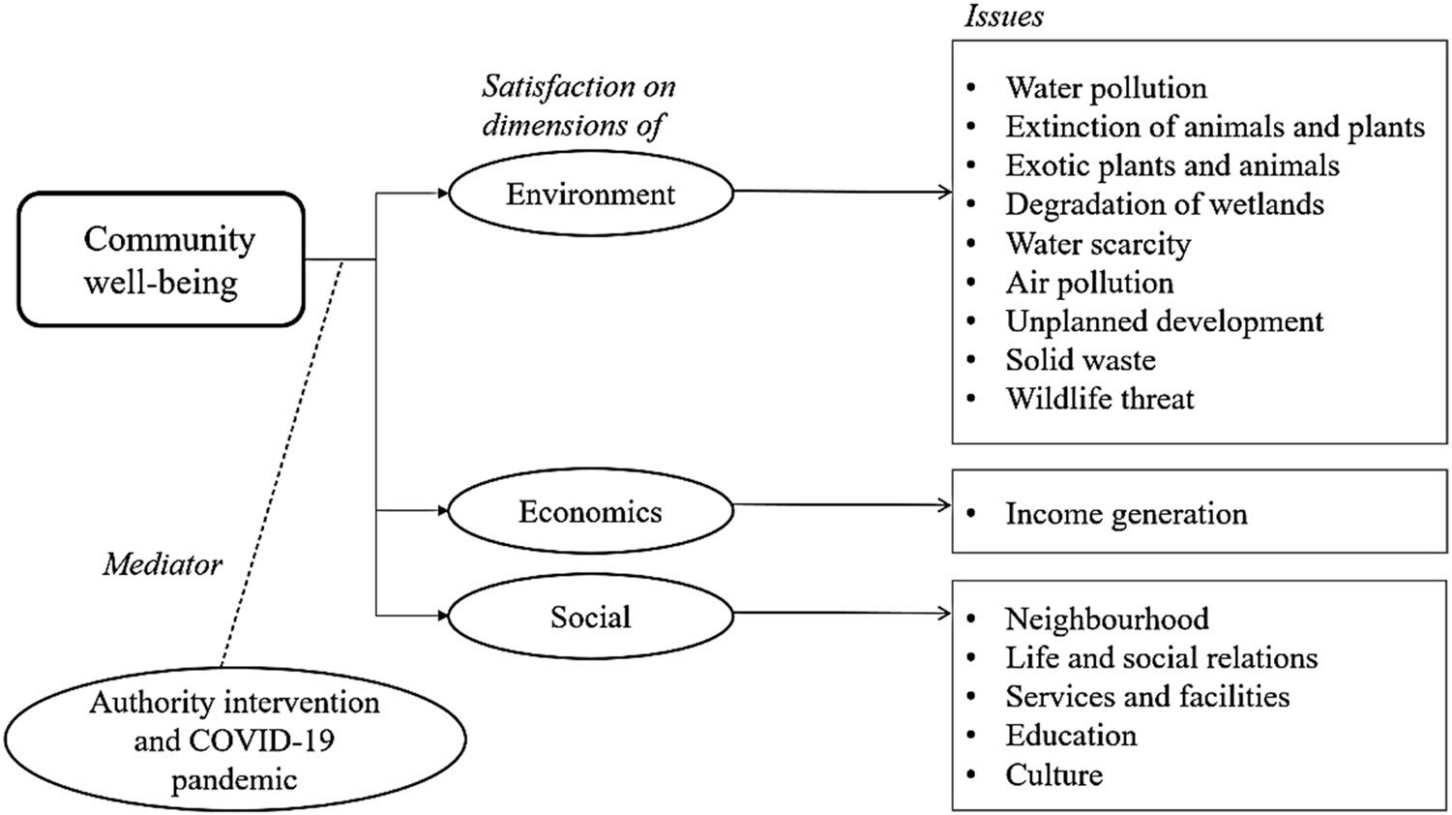
The conceptual framework used in the study. The community well-being is dependent on its members’ satisfaction with respect to environmental, economic, and social factors, as well as mediators such as authority intervention and the COVID-19 pandemic.

The conceptual framework was developed based on the relevant literature reviews on community well-being aspects that have been described previously. Figure 1 shows the conceptual framework built in the study, which covers the environment, economics, and social aspects with an authority intervention and COVID-19 pandemic as a mediator. The pandemic has affected the social community by forced them to isolate and disturbed the economy of society. It also caused a major changes toward environmental with the increase of domestic waste (Sharma et al., 2020). Thus, this study has to consider the pandemic Covid-19 as one of the mediators other than authority intervention in the well-being.

## Methods

### Research area

**Fig. 2:**
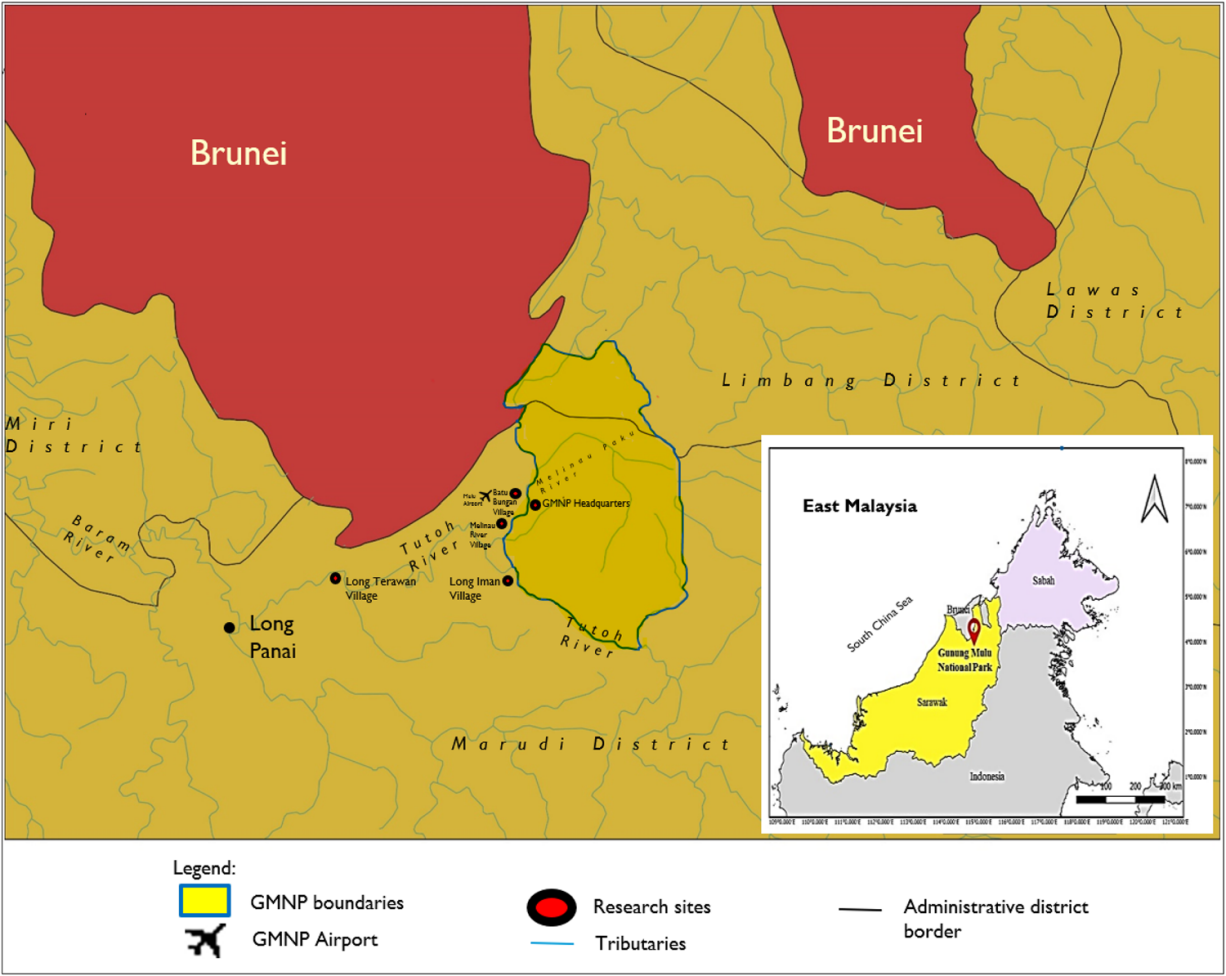
Location of GMNP, Sarawak, Malaysia. The map shows the distribution of research sites (red dots), which are GMNP Headquarters, Batu Bungan Village, Long Iman Village, and Long Terawan Village, which are inhabited by the Penan and Berawan communities.

GMNP is a national park located in Marudi Division, Sarawak, Malaysia (Fig. 2), which is one of the UNESCO World Heritage Site. UNESCO (2021) has clarified the WHS is the name given to locations on Earth that have exceptional importance to humanity as a whole and to be preserved for current and future generations to enjoy and appreciate. To date, 1007 natural and cultural places inscribed on the list such as Taj Mahal India, Grand Canyon (USA), Pyramids in Egypt, etc. This designation of WHS for GMNP is particularly beneficial for in-situ biodiversity conservation where greater awareness on its status could lead to the rise of level of preservation of its valuable properties. With the status, the areas under the WHS will be aided with financial and expert advice from the WHS Committee to ensure its sustainability of the sites. The given status also had improved community well-being in GMNP by allowing people to work together in enhancing their economic and cultural development especially through heritage tourism.

This GMNP area covers about 52,864 hectares of the mountainous part of northern Sarawak. It is separate from other developing areas, which lies between the headwaters of the Tutuh River and the Medalam River, a tributary of the Limbang River. Its location along the Brunei-Sabah-Sarawak-North Kalimantan Transboundary Landscape is one of the six priority landscapes in the protected areas of Borneo (WWF, 2017).

GMNP and nearby villages such as Sungai Melinau Village, Batu Bungan Village, Long Iman Village, and Long Terawan Village are inhabited by the majority of Penan and Berawan communities, which are indigenous to the park. The local community of Sungai Melinau Village from Berawan community is the most engaged in tourism services such as homestay, and transportation (e.g., longboat, and car) in Mulu. Other local communities mostly work as farmers or, fishermen for their livelihood.

The number of tourist arrivals to GMNP in 2019 is 21,022 and it is higher than in 2015 which was 18,632 (Sarawak Forestry Corporation, 2020). The trend shows that the number of international tourists is almost double that of domestic tourists. However, since the pandemic hit in 2020 and 2021, the data is not available for disclosure by the Sarawak Forestry Corporation due to the tourism industry’s progress being too slow.

### Research technique

This study employed a mixed-methods approach, particularly a concurrent nested design that is more appropriate by considering the time constraints and comfort of the respondents during data collection. This design gives priority to one of the methods and guides the study. While another is embedded, which aims at one of the methods, i.e., quantitative or qualitative method, and guides to address a different question than the dominant one or to seek information from different levels. The quantitative measures were triangulated with key informants’ narratives, which allowed for a greater understanding of the meaning of the quantitative findings (Cresswell, 1999; Teddlie and Tashakkori, 2009). Mixed methods that combine qualitative and quantitative data provide a convenient method of everyday problem-solving (Tashakkori and Teddlie, 2010).

Based on Miri Resident District Office (2020) a total population of Mulu Subdistrict is 4696. Using sample formula by Kothari (2004) with an acceptable error of 10% at the 95% confidence level, we consider to interview 99 respondents. With the limitation of movement due to COVID-19 pandemic, obtaining a sample size with a 5 to 10% margin of error seems impossible because of low number willingness to participate and fear of having contact with researchers. Kothari (2004) also emphasized that the selection of a research design for a sample size must be realistic, taking into account the budget and time constraints, and it is best to minimize sampling error as much as possible. Thus, the Likert scale questionnaire was disseminated to only 99 local communities in April 2021 through convenience sampling. The sample size is appropriate with an acceptable error of 10% at the 95% confidence level. The local community involved are those who live in the settlement areas around GMNP, including Kampung Batu Bungan, Kampung Long Iman, and Kampung Long Terawan. The respondents were to be from local communities who are more than 18 years old and live at the study site for more than 5 years.

For the qualitative approach, personal interviews with twelve key informants were conducted using snowball sampling. They were identified as key informants due to their first-hand knowledge and active involvement in the community. Their narratives provide a qualitative aspect that has meaning, significance, and rich understanding (Tashakkori et al., 2020). The number of key informants (*n* = 12) is acceptable for the qualitative approach as its nature requires a small sample size. According to Hammarberg et al. (2016), a large sample size is not recommended because it will possibly cause excessive data issues that will affect the depth of the scope for rigorous analysis. The key informant voices help to achieve data saturation, external validity, and/or information redundancy (Onwuegbuzie and Leech, 2007). This techniques of data collection are aligned with stated in Lietz and Zayas (2010) in increasing the trustworthiness of qualitative research. This qualitative longitudinal research method was chosen for this study because it is suitable for pandemic or disaster-related studies in which unique and rapidly changing environments necessitate more comprehensive descriptions of human condition (Terzis et al., 2022).Furthermore, we also employed the reflexivity method of writing memos or field notes throughout data collection in order to comprehend the significance of each interview and observation session as suggested by (Yong et al., 2019) to ensure the quality of our qualitative study.

Table 1 presented to show the characteristics of twelve key informants interviewed in different sessions.

**Table 1:** List of key informants involved in in-depth interviews in different sessions (*n* = 12).

Key informant	Category	Study site	Gender	Occupation
K1	Professional	GMNP Headquarters	Female	Research liaison officer, GMNP
K2	Professional	GMNP Headquarters	Male	Sarawak Forestry Corporation officer (Wildlife)
K3	Local community	Batu Bungan Village	Male	Tribal Chief
K4	Local community	Long Terawan Village	Male	Tribal Chief
K5	Local community	Sungai Melinau Village	Male	Homestay operator
K6	Local community	Sungai Melinau Village	Female	Homestay operator
K7	Local community	Long Terawan Village	Male	Park guide (freelance)
K8	Local community	Long Iman Village	Male	Boatman
K9	Local community	Batu Bungan Village	Male	Boatman
K10	Local community	Sungai Melinau Village	Male	Farmer
K11	Visitor	GMNP Headquarters	Female	Teacher
K12	Visitor	GMNP Headquarters	Male	Active hiker

### Data analysis

The descriptive data, which includes sociodemographic and respondents’ community well-being, were analyzed using the IBM Statistical Package for Social Sciences (Version 24). While the key informants’ narratives were themed deductively using Atlas.ti version 8 software. The themes highlighted the environment, economics, and social aspects, which play a crucial role in the community well-being in GMNP.

## Results and discussion

### Sociodemographic

Table 2 shows the demographic background of the respondents. Approximately 60.6% (*n* = 60) of respondents are male, while the remaining 39.4% (*n* = 39) are female. The majority of respondents (67.7%) are *Orang Ulu*, the Penan and Berawan ethnic who are indigenous to GMNP. Most of them have received at least secondary education and are employed and engaged in tourism services such as accommodation and transportation (e.g., longboat and car) in Mulu. Their income in the tourism sector was less than MYR2500 per month, considered the low-income group in Malaysia (Department of Statistics Malaysia, 2020).

**Table 2:** The demographic background of respondents.

Variable/Item	Local
	Frequency (%)
Gender	
Male	60 (60.6)
Female	39 (39.4)
Race	
Natives (e.g., Malay, Sarawak, and Sabah natives)	94 (94.9)
Chinese	4 (4.0)
Indian	0 (0.0)
Others (e.g., Non-Malaysian which include Caucasian, Bruneian, Indonesian)	1 (1.0)
Ethnics	
Penan	22 (22.2)
Berawan	45 (45.5)
Others (e.g., Malay, Iban, Chinese)	32 (32.3)
Marital status	
Married	73 (73.7)
Single	24 (24.2)
Others (e.g., divorced)	2 (2.0)
Age	
19–25	7 (7.1)
26–30	13 (13.1)
31–39	25 (25.3)
40–50	20 (20.2)
More than 50	34 (34.3)
Level of education	
No formal education	7 (7.1)
Primary education	20 (20.2)
Secondary education	58 (58.6)
Tertiary education (e.g., Ph.D, Master, First degree, diploma, certificates)	14 (14.1)
Occupation	
Government servant	14 (14.1)
Private employee	24 (24.2)
Self-employed	42 (42.4)
Retiree	2 (2.0)
Student	2 (2.0)
Unemployed	15 (15.2)
Monthly income	
Less than MYR2500	83 (83.8)
MYR2500-MYR4849	13 (13.1)
MYR4,850-MYR7,099	0 (0.0)
MYR7,100-MYR10,959	0 (0.0)
More than MYR15,039	0 (0.0)
Others (e.g., Prefer not to say)	3 (3.0)
Number of dependent	
None	41 (41.4)
1–3	40 (40.4)
4–7	16 (16.2)
More than 7	2 (2.0)
Observations	99 (53.2)

MYR1.00 = USD0.23 (Based on currency exchange in July 2022).

### Locals’ perspectives on well-being

Table 3 shows the mean analysis of community well-being based on the respondents’ perspectives. In the context of this study, community well-being is assessed through their satisfaction with the dimensions of well-being in terms of the environment, economy, and social perspective.

**Table 3:** Mean analysis of community well-being based on respondents’ perspectives (*n* = 99).

Pillars	Mean	Std. deviation	Satisfaction level
Environment	4.39	0.806	Good
Economic			
Income	2.46	1.264	Poor
Social			
Neighborhood	4.44	0.823	Good
Life and social relation	4.43	0.797	Good
Services and facilities	3.26	1.389	Moderate
Education	4.08	1.007	Good
Culture	4.09	0.882	Good

The average respondent is very satisfied with the environmental and social aspects of this GMNP. However, it was noted that they showed a moderate level of satisfaction with services and facilities. Although both the environment and social variables on average show good satisfaction among the respondents, they are not satisfied with the current monthly income, due to the COVID-19 pandemic, which is quite limited.

The well-being of the community in Table 3 is based on the respondents’ perspective. According to Ibrahim et al. (2021), the locals’ perception of elements related to well-being that are affected by governance is important and needs to be taken into account towards sustainability in general. Efforts to improve this ability need to happen at the macro level, which is the existence and ability of an organization to provide sufficient investment to empower individuals toward a sustainable community (Zamhari and Perumal, 2016). Consequently, twelve key informants clarify the element of well-being through the environmental, social, and economic dimensions.

### Environmental dimension

The mean analysis in Table 4, shows that the respondents state that biodiversity issues, as a whole, are small in the area. This includes water pollution, extinction of animals and plants, degradation of wetlands, solid waste, and wildlife threats. This is contradictory to the narratives given by key informants, including K1 in this study, who stated that these issues are something that needs to be paid attention to in GMNP and the surrounding area regarding wildlife conservation and waste management. It also gives a reflection that the local community has less awareness about biodiversity problems that occur in their area. The imagined environmental futures of communities illuminate significant issues within the existing relationships between themselves and their physical surroundings (Nash et al., 2019). Locals consider it a less significant matter, but it needs to be taken seriously.

**Table 4:** Mean analysis of respondents’ perspectives on biodiversity issues in GMNP (*n* = 99).

Issue	Mean	Std. deviation	Level of problem
Water pollution	2.00	1.229	Small
Extinction of animals and plants	2.30	1.388	Small
Exotic plants and animals	1.41	0.808	No
Degradation of wetlands	1.95	1.248	Small
Water scarcity	2.00	1.414	No
Air pollution	1.59	1.030	No
Unplanned development	1.43	0.928	No
Solid waste	1.96	1.301	Small
Wildlife threat	1.97	1.257	Small
Total	1.85	1.178	Small

### Water pollution

Tourism sector does not affect water pollution in GMNP. However, the attitude of a few parties who lack of environmental awareness contribute to the water pollution. For instance, private oil palm companies are reportedly conducting logging operations in the vicinity of GMNP, resulting in cloudiness of the river water in the region, particularly downstream of the river (Cheng, 2019). This issue seems to be beyond the people’s control because it involves local companies and authorities (Kendall, 2022).

Based on observations, water pollution involving toxic waste does not occur, but the cloudiness that has occurred in the downstream river side is due to the sedimentation of logging activities nearby the settlements.

You see how cloudy the river water is now? In the early 1970s, the Tutoh River was still clear and beautiful. We used to drink river water directly. (K10)

The logging companies are no longer operating in their area, but the cloudy river water remains probably due to domestic waste into the river by a few local communities.

### Solid waste

The issue of solid waste in the GMNP and its surounded arises as a result of several factors requiring attention from the facilicities management. Poor solid waste management, including the lack of facilities to treat it, will lead locals to dispose of trash in open spaces such as rivers (Salam et al., 2022).

There is no formal waste management system here. The locals have to manage their own waste. In the past, we used to propose a landfill, and the site was selected after a meeting with relevant stakeholders for many years. The population is increasing. The rubbish is increasing. I have experienced it myself in a longhouse where the people just throw the rubbish into the river that floats down. (K1)

The population growth in Mulu with the lack of services that are normally handled by the council is the garbage disposal. There is no proper sewage. Everything will be thrown onto the ground and eventually end up in the river. That’s why the river is polluted. It’s a major concern. We don’t have any policies on how to deal with the area that is outside the park but nearby. For example, okay, you can’t cut this. People just cut it, which means it can impact the microclimate and a lot of things. (K2)

Local peoples are more inclined to throw wastes into the river since long time. If this continues, it will degrade the water quality and directly harmful to the aquatic life. Next, the garbage suspended in the river will affect the scenic view of tourists during river cruising for visiting recreation area in GMNP such as Long Iman and Camp 5.

In the past, the locals usually threw leftover foods into the river because it was organic. That situation is acceptable, but now the current generation is throwing away plastic that won’t rot. (K8)

Garbage is dumped randomly into the Melinau River. There is a lot of plastic in the trees. (K10)

I burn garbage. If it’s a can, I dig a hole to plant it in. (K5, K6)

There are a few irresponsible people who throw garbage into the river, perhaps when there are no people in the river at night. (K6)

As locals who are custodians of this WHS UNESCO site, they suppose to have a better ecocentric attitude compared to visitors. However, based on K5, K6, and K9, the visitors are very disciplined, i.e., instead of randomly discarding trash. They pick it up from the side of the road and place it into the trash can provided at their accomodation. This may be due to the influence from the attitudes brought by ecocentric vistors to GMNP. Such visitors can influence locals to havebetter attitudes toward nature conservation(Arnberger et al., 2019).

The national park and Marriot Hotel have also worked together to run a community service program by emphasizing conservation-related environmental awareness education for the local population regularly, including river cleaning since 2006. To promote conservation activities, it is essential to have a well-developed, community-specific activity system including manpower, budget, community awareness, and consensus information (Hargreaves-Allen et al., 2017).

Normally, we try to organize things in a day. We divide them into different sections of the river. Penans from Batu Bungan Village will clean between here (GMNP headquarters) and their settlement, while Sungai Melinau villagers (Berawans) will clean up to Kuala Melinau (Tutoh River) because of the spread of houses in between. They will take the bigger section because the community is bigger. Then, we send a boat to assist the community as well. Everybody will bring their garbage here, and we will count and monitor how much is collected each time. We have this communal work. At the same time, running the awareness mainly focuses on trying to convince people not to dump rubbish into the river, which is the best way to deal with it. It is quite hard when some people change their practices, but some ignore the advice. They still dump it. Some villagers (from the Melinau River area) go by boat and dump it in the Tutoh River. It seems like the practice is still there, but there have been some positive changes where the rubbish is not as bad as it was previously, even though income and the number of people have increased. Now, this is not just a local community. But the clinic, school, airport, and district office staff are here as well. We hope that there is some progress because the garbage problem in the river will never be solved. (K1)

According to K1 and K8, both parties were also given a budget by the government to manage the collection of residents’ garbage in GMNP Headquarters and Marriot Hotel and transport it by boat to the landfill facility in Marudi once a month. This is seen as a cheaper initiative than building a landfill in the national park itself. The pressure of an increasing population causes the problem of solid waste management become a matter that needs due attention from relevant stakeholders.

To empower the local community, the park management has also proposed to the government to provide allocations for transporting rubbish using boats handled by the local community themselves, because most of them own a boat. This is also able to improve their well-being through generating income while fostering an ecocentric attitude that exists from a good place attachment.

### Degradation of wetlands

The degradation of wetlands in Mulu is caused by anthropogenic activities, including deforestation recently downstream and upstream.

Maybe one of the biggest issues getting worse now is clearing along the riverbank of a stream because of the COVID-19 pandemic where most villagers from Batu Bungan Village have started farming for their livelihood. But the problem is that many have been clearing trees right to the riverbank, which could increase erosion. While this problem happened in the past, it was not as severe as it is now. But now, it is becoming very severe because nearly all the trees on the opposite bank are all being cut down. (K1)

According to K4, K9, K10, and K11, deforestation for palm oil plantations caused landslides along the riverbanks, particularly in the downstream region. Although it occurs outside the GMNP, it still has the potential to disrupt the local ecosystem, especially the park’s high-diversity area. Due to the potential for environmental damage, monoculture plantation activities should not be conducted within 80 kilometers of the park.

Based on Brockerhoff et al. (2017), monoculture plantations are more likely to have lower levels of biodiversity compared to their surrounding native forests. In addition, loss of soil productivity and fertility, disruption of hydrological cycles, risks associated with plantation forestry practices (e.g., the introduction of exotic species), risks of promoting pests and diseases, increased risks of adverse effects of storms and fire, and negative effects on biodiversity are all potential outcomes.

### Extinction of animals and plants

Based on Table 2, respondents believe that the extinction of animals and plants in GMNP is nearly non-existent. This demonstrates that, on average, they are less aware of changes in population viability. They must be concerned about issues that have the potential to lead to extinction.

I think the area of this forest is still large, so animals will not be able to easily become extinct. Monkeys are also still in the palm oil plantation area for foraging. (K5)

I think the issue of extinction is not there at all. (K6)

However, based on K7, K10, and K9, animals including pangolin and sun bear are increasingly difficult to see than before, and these animals are likely facing the threat of extinction due to irresponsible hunters in the GMNP area and its surroundings.

We eat most of the animals in the park, to the point of posing a threat to them. We usually hunt mice and deer outside the national park. Now it is almost difficult to see hornbills, pergam, punai, and kuang birds compared to the 1960s. Based on my personal opinion, Ulu people don’t love animals. Other animals such as forest cats, foxes, bats (large hawk-eagle), pythons, and red cats are also hunted. As for the trees, large and old trees were once cut down to be exported abroad. You can see the view of the forest that is diminishing from the air space when taking the plane here. The only remaining wood is small and young. (K10)

Limited awareness of extinction has also been documented in other rural communities surrounding protected areas (Ma et al., 2021). Nonetheless, every individual should be concerned about the extinction of these species. To protect the diversity of species in GMNP, it is necessary to consider the extinction-causing factors.

### Wildlife threat

The primary threats to wildlife in the area are hunting, deforestation, and oil palm monoculture plantations.

They have the right to hunt wild boar, deer, and mouse deer and fish in certain sections of the river. For the nomadic Penans, they could hunt in any area of the park, but there are few nomadic Penans left and most of them have settled. But we consider the people at Batu Bungan Village as semi-nomadic, and they go into the forest sometimes. They were given a certain area for hunting and fishing within the park. Basically, it has become a problem because they just live opposite the park and river, so they can easily enter the park for hunting and fishing. They do hunt and fishing outside the designated areas that are located next to their village. The problem is that hunting is not restricted to wild boars. Some people hunt endangered and even totally protected species. There are not many animals in terms of the abundance of mammals due to hunting. It is not just restricted to Penans, but some Berawans also hunt in the park. There is much less hunting on their side. There is more hunting downriver from here but not inside the park, particularly for wild boar. But it does not mean they did not hunt here. (K1)

In Mulu, locals living near the national park have the right to hunt wild animals that are not protected. But when they hunt, usually the people there will hunt anything they find. Although there is a law, the parties here are less able to carry out effective enforcement. (K2)

Frankly, I have hunted, and it is most likely in the picture (given pictorial questionnaire). But we do not hunt many animals at a time. It is just a few animals that are sufficient for the number of family members. We usually hunt monkeys and squirrels. Birds are hard to catch because they fly. I use a blowpipe that contains rubber poison to hunt. (K9)

Hunting, so far, is a lifestyle for the Penan and Berawan for survival. They do not keep animals for food because they have been trained to hunt and find fish as food since a long time ago. To forbid them not to hunt is quite impossible. They will eat all the animals. Furthermore, they use blowpipes, which are considered silent killer tools that can catch more animals and are not so easily detected compared to using guns. (K7)

Illegal wildlife trade is also likely to occur, involving locals and outsiders. K7 explained that pangolin species are in demand from buyers. He also explained that some use tissue culture to breed certain species of orchids and sell them quietly. Similarly with Phong Nha-Ke Bang National Park, Vietnam, where a few residents are not concerned about selling wild animals to outsiders for their profit (Truong, 2022).

Next, disruption of the ecosystem may occur due to the conversion of forest to a monoculture plantation. According to a study by Ridwan et al. (2011), the monoculture oil palm plantation in nearby areas will reduce the foraging and roosting activities of tropics bat species, which rely heavily on forests for food and shelter.

The park itself is well-protected. Maybe one of the issues hunting in the park is not a huge major issue. Other than that, there are threats to the area outside the park because the boundary does not protect all the species within the park because some species move outside the boundaries. One example is wrinkled bats in Deer Cave as you can see, they fly very far from the park which is exposed to threats outside the park that could affect their population, and one of the biggest problems is probably monoculture plantation outside the park. This is why UNESCO has recommended not clearing the forest or monoculture tree plantation activities within 25 km of the boundary of the park. But actually, there have been some issues with it as well. There have been some areas outside the park where it is designated for oil palm plantation and opposed by locals. (K1)

Logging for oil palm plantations is happening outside the park and I don’t agree. Imagine fitting into a palm oil plantation right next to this WHS. If the forest is all cut down, the animals will run to the untouched virgin forest of Brunei, which is not far away, only 21 kilometers from here. Here we are messed up. (K7)

Bat species in GMNP are keystone species because their extinction will affect the cave ecosystem. Guano from bats is an important energy source with large, varied, and unique ecosystems existing around such deposits (Moulds et al., 2013).

I think it affects the population outside of the park, for the forage would probably affect the future of the park, primarily because they forage in open areas and above the canopy. Now, they are probably dependent on insects above the canopy since insects are abundant. If the forests are cleared, there will be fewer insects, so they will lose food. If the area is cleared for monoculture plantations, they tend to introduce a lot of pesticides that will affect the bats. Not just bats themselves, because when they fly back to caves, they affect all the dependents and everything in the cave, so their adolescents will also be affected. (K1)

If you do a lot of things around GMNP, then things in GMNP will not be able to survive. Just in case, the outstanding universal value in Mulu is the swiftlets and bats in the cave, but the food source is only 50 km around the cave. That brings you all the way to Brunei or other parts of Baram, but if there is no control over land use in the area, there may be fewer food sources for these animals. So, the number of OUVs could be impacted, which is very critical. (K2)

Some Berawans, including K4 and K5, also stated that they always go to the GMNP forest area bordering Labi Forest Reserve, Brunei, and claimed that they can see more wildlife, including a maroon-leaf monkey and gray-leaf monkey species. It seems that the area is good to support the survival of the species due to the lack of human interference and provide foods and shelter. This area is located in the Heart of Borneo priority landscape, which is the Brunei-Sabah-Sarawak-North Kalimantan Transboundary Landscape, which acts as an ecological corridor that connects wildlife, including endangered species such as Borneo orangutans, Borneo pygmy elephants, hornbills, and Muller’s gibbon, among others, that thrive in the region (Keong and Onuma, 2021).

However, not all animal species have migrated towards Labi Forest Reserve, as claimed by residents. This is because GMNP is a habitat that has the most suitable ecosystem for several species, including bats.

Obviously, the park hosts a lot of wildlife, like quite a few protected species, especially those related to caves (which feed in caves, etc.). It depends on the species and what kind of habitats they have, such as limestone, because they can’t go to Brunei. After all, there’s no limestone on the Brunei border. Even the karst itself provides the surface with cavities, which provides space for animals to hide, especially mammals. There’s no support area in Brunei for them. Brunei, of course, has a forest, but it does not have karst and cavity caves that are found. Mulu has karst to support it. Higher elevation plays a crucial role. Brunei does not have high mountains, so many species are also restricted to higher altitudes. We here have a lot of endemic species, especially frogs and reptiles, that just can’t go to Brunei because the habitat is not suitable there. (K1)

By observing the animal hunting that takes place in GMNP and its surrounding area, the locals are still subject to the National Parks and Nature Reserves Ordinance of 1998 and the Wildlife Protection Ordinance of 1998 even though they have certain rights as indigenous people, including being allowed to hunt certain animals. Traditionally, local people hunt animals in this such areas for their livelihoods, but these local hunting restrictions cause them to slightly change and tend to depend on governments and other NGOs to provided financial aids (Heim and Pyhälä, 2020).

### Economic dimension

The COVID-19 pandemic has caused a significant change in the locals who have been heavily dependent on the tourism sector for survival. K3 and K4 feel proud because they live in the main area of world-class tourism, which is GMNP, and many benefits they receive are due to the existence of tourism. It changes their quality of life via income generation opportunities.

We are proud because we have GMNP. There are job opportunities, and there are also tourists coming from Miri and Marudi by boat and staying here (Long Terawan Village) for a few days before going to the GMNP. (K4)

Since the outbreak of the pandemic that caused economic paralysis, however, most respondents have become dissatisfied with their monthly income. Before the pandemic, the majority of the local community had earned a t minimum of MYR2,000 (USD442.48) per month. However, as a result of the pandemic, the income has decreased to less than MYR1,000 (USD221.24) per month. The situation forcing them to engage in gardening, fishing, animal husbandry, and other small-scale agricultural activities despite lacking agricultural expertize for their survival.

Our income has been bad since the pandemic. I don’t dare to open a homestay either. (K5)

I experienced hardship because there were no tourists, no work, no money, and I just stayed at home while working on small gardens. (K3, K6, K8)

It is not only the self-employed who are affected by the effects of this pandemic. In fact, according to K1, the number of staff was also reduced from various positions, including those working in the café, housekeeping, and some of the park guides.

The COVID-19 pandemic has taught everybody and given lessons on not taking everything for granted. People began to realize how much money they could make from tourism. Things shift. You can see most people are farming now, which is different from their previous lives, where most of them are engaged in tourism. For example, people in Batu Bungan Village who work as boat operators, handicraft sellers, park guides, or freelance park guides have lost their income. But now they are turning back to where they were years before the park opened. They go to the farm. Even here, there is no market for people to buy food unless people sell to each other what they grow. The shop is also empty nearby nowadays. Not much there. They have twice-daily flights from Miri, as previously stated, so some will bring frozen foods, chicken, and meat. For the past few months, we’ve just had one flight, which is nothing convenient. People just depend on fish and what they grow. (K1)

In addition, a similar situation occurred at the Marriot Hotel, where an estimated 80% of staff (100 individuals) were laid off due to current financial constraints. The hotel had to spend approximately MYR150,000 (USD33,673.83) on maintenance, particularly the electric generator, despite the low number of guests. Next, the local community faces the challenge of modifying their way of life to accommodate the pandemic.

As if there is no other alternative, if you want to market handicrafts to outside areas, you need a fast postal service, and it is quite difficult to do so because the location of the post office is quite far. Even if you run an online business, you still need the internet, which is a broadband network, which is limited here. (K3)

Now, young people are more stressed because many have lost their jobs. They only rely on freelance work. There used to be tourists who could use boats to earn an income, but now there are none. It’s really difficult because of the pandemic that has been going on for more than a year now. We do a little business to cover our needs at home, such as selling drinks, cigarettes, and fried chicken. We thought of going to Miri many times due to having to follow procedures, including applying for a permit across the area, which is quite harassing. (K6)

Changing jobs is very difficult for me, who is used to tourism. Even if you want to start a business, you still need a lot of capital. (K7)

Our income is uncertain. We also look for umbut (the soft root of edible palm trees) and sell it to the villagers. (K9)

Based on K12, there is a freelance park guide who has been laid off and has been able to generate income through his own YouTube account platform. The content of the uploaded videos revolves around his life during the pandemic, and it was acknowledged by K12 that he has good video editing skills and his presentation about geology in GMNP is very easy to understand by the audience. In conclusion, the diversification of livelihoods in GMNP in terms of different types of jobs is lower, which means that almost all of them depend on the tourism sector compared to others. Therefore, it is anticipated that the level of locals’ adaptability to changes caused by this pandemic will be low. According to Makwindi and Ndlovu (2022), diversification is the most important strategy for surviving economic pressures caused by natural disasters such as this pandemic, which affects the income of the majority of people. All parties must understand the risk and vulnerability of relying on a single major source of income, as the COVID-19 pandemic has a significant impact on livelihoods (Smith et al., 2021).

### Social dimension

Despite their differences in political ideology, the majority of respondents believe that neighborhood life and social relations are still positive. The concept of togetherness is still practiced among them through cooperative activities to collect garbage around the village and river periodically through a program organized by GMNP and Marriot Hotel management based on K3, K4, K5, and K8. Community involvement is a crucial indicator of the success of protected area management (Wibowo et al., 2018). While visiting neighbors and sharing food is still a common practice among them.

We also shared a wild boar (hunted animal). If they don’t share it, they will buy it with us at a low price. (K4, K5, K9)

Due to tourism, there are also locals, particularly from the village of Long Terawan, who marry tourists or foreign workers. This marriage, which represents their acceptance of foreign culture, is viewed favorably, and it is a reflex that they are adaptable in accepting positive changes such as education and employment.

On the contrary, the local community is also very satisfied with the education received by their children since the 1990s, even though the location of the secondary school, which is Long Panai Secondary School, is quite far from the GMNP area and it takes almost 2 h by boat.

The reputation of the school here is very good. Some former students got excellent results in Malaysia Education Certificate (SPM), i.e., 9A, and managed to continue their studies at university. (K10)

### Cultural heritage

The level of satisfaction of respondents towards culture is good (Table 2). In terms of traditional dances and musical instruments, residents still practice them intact to be presented to dignitaries and tourists who come here as tourist products. Next, the making of handicrafts and other forest products by the Penan community shows that these skills are still in good condition.

Although cultural heritage is not viewed as a major issue by the study’s respondents, who are mostly young and middle-class. However, some narratives in the study explain that there are still issues related to intangible cultural heritage, especially in Batu Bungan Village and Long Iman Village, which is in the Penan’s Oroo’ language.

Oroo’ is a language commonly used by previous generations of Penan for communication purposes in the forest such as navigation using signs from tree twigs and leaves. (K1)

The arrangement of the twigs and leaves describes the combination of words in a sentence. The message can be translated if the individual understands each word that is trying to be conveyed. Oroo’ is the object of writing language used by earlier generations to leave messages for each other in the jungle (Jensen, 1970). Sticks, prepared with cuts, twigs, and leaves in certain positions and places, will guide people, and inform them about directions, time, dangers, resources, etc. (Rothstein, 2020).

According to K3, the Oroo’ language is only part of the customs and culture and can still be understood by many people, especially the older generation. However, the language is poorly understood among the average younger generation. It is because they have received a formal education in school and can read and write well. Thus, the importance of mastering the Oroo’ language has become less significant for them. Furthermore, the language is rarely used and is considered an ancient language. Similarly, based on a study by Plimmer et al. (2015), it was found that the language has disappeared and is no longer used by the younger generation among Penans in Long Lamai since they were settled.

According to UNESCO (2011), the loss of indigenous languages is also detrimental to biodiversity, as traditional knowledge of nature and the universe, spiritual beliefs and cultural values expressed in indigenous languages provide time-tested mechanisms for the sustainable use of natural resources and management of ecosystems. These elements have become more critical with the emergence of urgent new challenges posed by climate change.

**Fig. 3:**
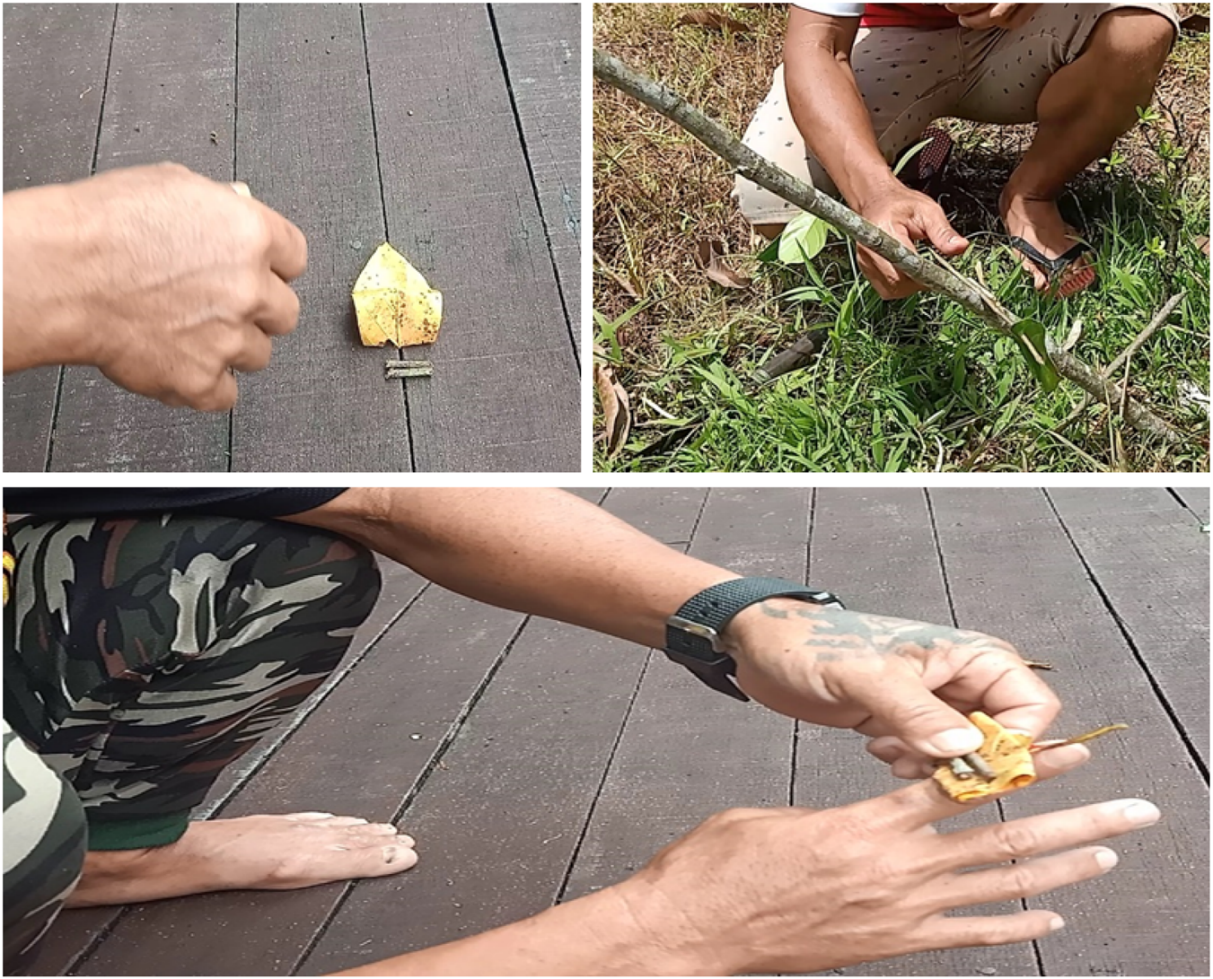
The basic Oroo’ language explanation by key informant. It indicates that the Penan community uses twigs and leaves to form specific signals that carry certain messages. The symbol of two twigs of the same length indicates the presence of a team/ one/ friend/ family (non-enemies) while in the forest. The two twigs placed on top of the folded leaves indicate hunger signals to non-enemies (friends, family, acquaintances). While the combination of the symbol of a leaf pricked by a small twig, then inserted with a tree branch carries the meaning that the individual has obtained the hunted animal (wild boar or other foods) in this straight direction.

Figure 3 shows K9 explaining the basic Oroo’ language which indicates that the Penan community uses twigs and leaves to form specific signals that carry certain messages. This clear explanation shows that the middle-aged generation is good at the Oroo’ language. It further supports the statement of K3 that the middle-aged generation can still understand the language very well as compared to the young-aged generation (born in the 90 s or later). It is in line with Zaman and Jengan (2014), where the respondents over the age of 60 have mastered the language because they experienced a nomadic life when they were young (Zaman et al., 2014).

K9 stated that he had learned the Oroo’ language through experiences with his father when hunting in the forest and looking for sago since childhood. He was accustomed to wading through the forest for that purpose and work of cutting and carrying trees and removing obstacles along the way in the forest.

We did not have formal classes to learn this Oroo’ language. We are indirectly good at using that language. (K9)

However, according to K9, some Penan’s younger generation who live with their families and semi-nomads in the jungle, are still able to use the language compared to the community in Kampung Batu Bungan.

My 11-years-old son is already good at using sumpit (blowgun). He is good at using poison for hunting purposes because he always follows me to the forest, and I taught him. (K9)

In conclusion, this informal lesson has the most crucial value in the Penan community. The Oroo’ are themselves expressions of social interaction. Given the fact that people usually travel together, the reading and interpretation of the signs is also a social practice, although often the meaning of Oroo’ is rather explicit (Rothstein, 2020).

Most young people do not understand Oroo’ language, especially those born in their 90 s and above. (K9)

The elders realized that Oroo’ will be lost if they do not find ways to preserve and pass it through to the younger generations (Plimmer et al., 2015).

K9 also informed that their previous lives were more difficult, and the younger generation is having lack of interest to go into the forest. Some were afraid to go into the woods, unlike the middle age who had experienced and were trained for it.

Furthermore, as mentioned by K3 earlier, words have become the primary medium of communication in society nowadays, and it affects the Oroo’language to be less important. The times and lifestyles slowly changed, and they adapted to the new situation. Indirectly formal education and socioeconomic factors caused this transformation to take place.

Due to the pandemic, the tourism sector has stagnated for a while. As a result, local people have lost their source of income due to the lack of tourists. Furthermore, K3 also stated that a handful of villagers are chosen to stay in the forest to avoid COVID-19 virus infection. Penan community in Kampung Long Iman hid and ran away from home when medical officers came to their village to perform a COVID-19 polymerase chain reaction swab test (Nais, 2021).

The increasing number of COVID-19 infection cases everyday is likely to cause many individuals to move to the forest. On the positive side, they can spend time together in the woods. This further strengthens the family bond and indirectly, they engage in the traditional lifestyle that was practiced by the earlier generation. The young-aged generation can have the opportunity to learn and experience the Oroo’ language from the middle-aged.

### Services and facilities

The locals recognize that the level of services and facilities here is average and that it still requires significant attention from stakeholders, particularly concerning water and electricity supply.

The electricity facilities here are bad. On average, we still use our generator. Some villagers use solar. I also have five water tank units and one of them is given by the government. (K5)

We do have problems with our water supply. I live on the side of the road (far from the river) and only rely on rain catchment water. If it’s a dry season, we don’t have water. There is no clean water supply (treated water) in this Mulu area. Only the national park and the Marriot Hotel have treated water supplies because they have filters and chlorine. Those of us who live in single-family homes do not have that water. Those who live by the river also use engines to pump water into their houses. (K6)

They are aware that the available river water is not necessarily safe to drink due to various factors. Clean water and electricity are basic needs for human well-being, and everyone has the right to have access to clean water which is in line with Sustainable Development Goals 6 (Purba and Budiono, 2019).

### Authority intervention as a mediator

The well-being of the community also depends on the authority’s role in determining their quality of life in principle. In the context of tourism, the authorities have the power to mediate its intensification, form a policy, and determine the parties that should benefit through the implementation of the policy (Zinda, 2017). Holistic management by the authorities will encourage the local population to support the implementation of the policies carried out in this GMNP. This is in line with the findings of Park and Inanç (2017) that the positive behavior of locals towards conservation in protected areas is dependent on the current management strategy that involves local communities more effectively. Despite this, their dissatisfaction with an effort that the government is trying to implement needs to be taken into account and resolved through consultation. It is noted that there are several issues involving the authorities, especially regarding road proposals and community conflicts in the area, although the government helps a lot from an economic point of view.

### Road proposal

The Sarawak government has proposed new roads linking Miri-Marudi, Marudi-Mulu (Kuala Melinau), and Long Panai-Long Lama under the High Impact Infrastructure project. The project will increase accessibility from Mulu to other areas. Although this project makes it easier for locals to reach facilities such as hospitals, schools, and grocery stores in Miri at any time. However, many do not support the project due to some challenges that will arise as a result of the development.

It’s not that we don’t want to, and it’s not that we really want to. Indeed, since long ago, there has been a trail or unpaved road that connects this Melinau area. It starts from Long Iman-Long Lama-Long Bedian-Miri. I don’t want a paved road directly from here (Melinau) to Miri. The land we have now is not big enough anymore. It has already been invaded, and such development will only make us more trapped. (K3)

In my view, it is enough as it is now. There is no need for a road. (K9)

This proposed road will not only put pressure on the locals of Batu Bungan and Long Iman Village, but it will also affect the economy of those from Long Terawan Village. This is because water transportation from Miri to GMNP will be paralyzed due to the existence of the road. All this time, the residents of Long Terawan have earned a decent income from rural businesses that have been welcomed by international tourists. The village becomes a transit point for tourists to experience longhouses and mingle with the residents. Thus, residents can also sell local products such as rice wine (an alcoholic drink), dried fish, and other agricultural products. The income of boat operators will also decrease as a result of the construction of the road. Therefore, looking at the impact that will happen, it is better to maintain air transportation as the main transport to this GMNP where the locals are indeed given a subsidy for the cost of their air ticket from Mulu to Miri Airport, which is considered reasonable.

In addition, the construction of the road is likely to result in even worse degradation of biodiversity.

An increase in population will also occur due to the existence of roads that connect Mulu to other areas. Pressure on the use of natural resources in Mulu will also occur, leading to encroachment, hunting, pollution, and so on. (K2)

What is the guarantee that the parties involved in the construction of the road will not take timber when carrying out the project? Make sure they do the construction work without damaging the existing environment, which may threaten us. (K10)

While first in protecting the park and biodiversity, the road connection to Miri and the rest of the area is a terrible idea. It will mostly destroy the biodiversity because it will make it easy for outsiders (wildlife traders) to come in and hire locals to collect in the forest and transport it directly. The wildlife trade is a big problem for us. Even though the local community hunts and the park is primarily for subtenants, they are not involved in the wildlife trade. Most local communities are also against the proposed road because they worry about the competition from outsiders coming in. Now, they monopolize transportation around here. Although people favor the road to Mulu, but not directly to here (consider at some stages). It should be because it directly limits how people come. One idea to solve this problem is to have the end of the road be at the Tutor River. Then people will travel the rest of the journey to Mulu by boat. It has frequently been discussed among the Berawan community about this. Many residents support the road, but they are concerned about encroachment on the park. They are aware of the effects of tourism on their livelihood because they are involved in it. (K1)

This demonstrates that locals are highly concerned about potential threats to biodiversity in GMNP and the surrounding area posed by road construction. Despite opposition to the proposed road construction, it was the authorities who determined the most effective means of controlling the situation to ensure the well-being of the community. The authority should note that locals can be a direct threat to the protected area when they refuse to cooperate with them or participate in conservation activities (Holmes, 2013).

### The government’s assistance in empowering the local economy

Creating a balance between park protection and community development is a global challenge for national park policymakers and management authorities (Peng et al., 2022).

In terms of the economy, the government also provides a lot of assistance to tourism operators, including homestay operators who emphasize human capital. According to Croes et al. (2018), investment in human capital is the key to sustainable tourism development through good hospitality to tourists. In GMNP, some locals are given courses and facilities to operate business-related tourism by local authorities.

Last year, some officers from the Sarawak Economic Development Corporation came here to inspect the state of our homestay. They monitor if there is a lack of facilities such as washing machines, beds, refrigerators, and beds. If before, they gave us MYR5000, but now the who bought us the appliance corresponded to the value. (K6)

There are also homestay-related courses given by the government in Miri. (K5)

### Land ownership conflicts

The issue of land ownership among the Penan-Berawan people around Mulu has been a matter that has arisen recently, causing the government to intervene in finding a proper solution.

It can be said that there is an encroaching party like the Berawan people who came to our place and claimed that this land is their right. Their border is far away from here. While they have a village, they don’t live there. That is the issue that I want the Chief Minister (CM) to solve because they are still very much in power (dominant) here. In terms of work, it’s not a problem. Recently, they requested the Chief Minister to build a longhouse. Once, the CM approved the construction of our longhouse here, and he was attacked by a few of them. This is a bad experience. (K3)

As far as we are concerned, we are not worried. Due to the presence of immigrants from other villages and races, we are worried. I do not agree with the development. Right now, our land is an issue. We imagine our land will be taken away. Where else do we want to live? Now the Chief Minister is going to Marriot Hotel to discuss this land issue. A few Berawans claim that this place where we live is their land. Even though we have always lived here. (K9)

Although ethnic conflicts have existed for centuries, However, they remain united in their opposition to forest destruction in their region, which shows their place attachment is good. Based on Zhang et al. (2020) and Mohamad Syahrul Nizam et al. (2021), place attachment refers to an emotional bond which is a memory produced through experience in an area and it plays a role in fostering an ecocentric attitude.

UNESCO WHS status helps here. If there is no such status, they, namely Radiant Lagoon Sdn Bhd, are already producing palm oil in the area near here. We (Penan and Berawan) drove them away. People who have a stake in Radiant Lagoon Sdn Bhd do some business here. They take opportunities (tricks) when running some businesses here, including attempts to exploit the area, which is only seen to affect the environment and not to the detriment of the local population. (K10).

The study carried out by Brankov et al. (2022), Eben (2006), Mannetti et al. (2019), Nastran (2015), and Ngonidzashe et al. (2017) also stated the same situation, which is the conflict between locals and protected area management. Although it involves different issues from different groups, it has similarities in terms of dependence on natural attributes in national parks.

## Conclusion, caveats, and policy implications

The well-being of the community through their satisfaction with the environmental, economic, and social aspects of GMNP has been unraveled, and some of the issues that have arisen have also been explained in detail by key informants in this study. In general, the local community’s perception of the environmental aspects of GMNP is good. However, it does not reflect the actual situation, i.e., river water turbidity, wildlife threats, degradation of wetlands, and solid waste issues still occur, especially in areas at least 50 kilometers outside of GMNP. Next, the constraints of the COVID-19 pandemic show a decrease in their income. For survival, they changed to gardening, fishing, hunting, and small-scale agriculture activities for continuous food supply. In terms of social aspects, the services and facilities are needed to be improved, especially the supply of clean water and electricity. Some of the local communities are very satisfied with the cultural aspects of their lives, which include traditional elements such as handicrafts, dance, and musical instruments. Based on the narratives, the Oroo’s secret sign language of Penan, which is their identity, is increasingly threatened due to changing lifestyles and the need for documentation and promotion of tourist products for the language. Authority intervention also greatly affects the well-being of local communities due to policy implementation in terms of environmental, economic, and social aspects. Local support towards the government can determine the holistic management of biodiversity conservation and sustainable tourism in the natural area like GMNP.

Therefore, the bottom-up approach is seen as an idea that should be emphasized by stakeholders, which is the involvement of all parties in the decision-making process starting from the ground level, and it is important in understanding the well-being of the complex community. Communication involving all stakeholders can determine the form of community empowerment and lead them to support a balanced form of management. The holistic management in a protected area, particularly UNESCO World Heritage Site will provide good periodic reporting, which is a reflex that the site is well-managed by stakeholders and there is no significant threat that makes the site listed on the List of World Heritage in Danger. The elements of biodiversity conservation and well-being are among the emphasis in the periodic report which is carried out every six years by appointed assessors. In particular, the process involves an assessment of the detrimental elements of a property, whether its condition is stable or not, and the UNESCO World Heritage Committee will provide recommendations based on the report. In addition to the global branding as WHS, any protected area also has the potential to be proposed as one of the IUCN Green List of Protected and Conserved Areas if the components include good governance, sound design and planning, effective management, and successful conservation outcomes in good conditions.

The situation in GMNP explains that the local community has met certain well-being criteria based on definitions from various literatures that explain the relationship among the environmental, social, and economic domains. The social psychological aspect demonstrates that it is the key in achieving well-being. Finally, place attachment is interpreted as a catalyst for a resilient community in GMNP. This study has highlighted the challenges posed by the pandemic to a tourism community residing in one of the protected UNESCO World Heritage Sites. The empowerment of local communities constitutes comprehensive conservation of biodiversity. The conceptual framework of this study can be applied to other studies on related topics, such as the well-being of local communities in protected areas in both developed and developing countries. This mixed-methods study provides greater insight than a quantitative study alone into community well-being, environmental, economic, and social issues, the role of authority intervention, and the COVID-19 pandemic as a mediator. This is due to the fact that, in general, local communities living in protected areas have similar environments, i.e., they rely on biodiversity characteristics that may provide slightly different benefits and challenges based on their perspectives.

Future research will benefitted with broaden the scope of the study towards psychosocial aspects (awareness, knowledge, attitude, and experience of biodiversity conservation) and sociodemographic factors that may have an impact on the respondents’ own level of well-being. It is also recommended for the future research to explore the community well-being in terms of economic aspect, particularly the value of biodiversity conservation in GMNP. It is potentially, could provide a new context into the existing data that relates to human well-being.

Taking into account how challenging it is to conduct sampling during this pandemic, the mixed method approach can reduce the likelihood of bias in research findings. We acknowledged that this study has its data limitation, however it could represent a pragmatic and prioritize a meaningful knowledge on the application of quantitative and qualitative methods. Arbitration between these two approaches is crucial to understand the community’s perspective through the eyes of key informants and the generalizations of laymen. In addition, this study includes local communities residing in the isolated area who, on average, have a low level of literacy and limited internet access. Thus, it limits the ability of online surveys. Even though this mixed method is deemed appropriate for use during this pandemic, researchers must be aware that data reliability may be compromised. Thus, it is recommended that researchers engage in data triangulation to ensure its reliability by engaging in reflection that consistent with the study’s objective(s), epistemological stance, and design. In addition, the extent to which threats and biases in the study can be adequately managed.

## Data availaibility

All data generated or analyzed during this study are included in this published article.
